# OpenSense: An open-source toolbox for inertial-measurement-unit-based measurement of lower extremity kinematics over long durations

**DOI:** 10.1186/s12984-022-01001-x

**Published:** 2022-02-20

**Authors:** Mazen Al Borno, Johanna O’Day, Vanessa Ibarra, James Dunne, Ajay Seth, Ayman Habib, Carmichael Ong, Jennifer Hicks, Scott Uhlrich, Scott Delp

**Affiliations:** 1 Department of Computer Science and Engineering, University of Colorado, Denver, CO USA; 2 Center for Bioengineering, University of Colorado, Anschutz Medical Campus, Aurora, CO USA; 3 Department of Bioengineering, Stanford University, Stanford, CA USA; 4 Department of Mechanical Engineering, Stanford University, Stanford, CA USA; 5 Department of Biomechanical Engineering, Delft University of Technology, Delft, Netherlands; 6 Department of Orthopaedic Surgery, Stanford University, Stanford, CA USA

**Keywords:** Inertial measurement unit, Open-source, Kinematics, Biomechanical model, Drift

## Abstract

**Background:**

The ability to measure joint kinematics in natural environments over long durations using inertial measurement units (IMUs) could enable at-home monitoring and personalized treatment of neurological and musculoskeletal disorders. However, drift, or the accumulation of error over time, inhibits the accurate measurement of movement over long durations. We sought to develop an open-source workflow to estimate lower extremity joint kinematics from IMU data that was accurate and capable of assessing and mitigating drift.

**Methods:**

We computed IMU-based estimates of kinematics using sensor fusion and an inverse kinematics approach with a constrained biomechanical model. We measured kinematics for 11 subjects as they performed two 10-min trials: walking and a repeated sequence of varied lower-extremity movements. To validate the approach, we compared the joint angles computed with IMU orientations to the joint angles computed from optical motion capture using root mean square (RMS) difference and Pearson correlations, and estimated drift using a linear regression on each subject’s RMS differences over time.

**Results:**

IMU-based kinematic estimates agreed with optical motion capture; median RMS differences over all subjects and all minutes were between 3 and 6 degrees for all joint angles except hip rotation and correlation coefficients were moderate to strong (r = 0.60–0.87). We observed minimal drift in the RMS differences over 10 min; the average slopes of the linear fits to these data were near zero (− 0.14–0.17 deg/min).

**Conclusions:**

Our workflow produced joint kinematics consistent with those estimated by optical motion capture, and could mitigate kinematic drift even in the trials of continuous walking without rest, which may obviate the need for explicit sensor recalibration (e.g. sitting or standing still for a few seconds or zero-velocity updates) used in current drift-mitigation approaches when studying similar activities. This could enable long-duration measurements, bringing the field one step closer to estimating kinematics in natural environments.

**Supplementary Information:**

The online version contains supplementary material available at 10.1186/s12984-022-01001-x.

## Introduction

Inertial measurement units (IMUs) could enable biomechanics and rehabilitation researchers to measure kinematics in a variety of populations, in natural environments and over long durations. From detecting functional improvement in patients post-stroke to monitoring fall-risk in older adults [1], continuous sensing of kinematics could improve our understanding of human movement pathology by providing many repetitions of a movement in home or community settings, in contrast with the limited number of trials and highly-controlled environment of a laboratory experiment. IMUs could also enable early detection of disease or injury-risk. They could then be used together with mobile interventions to create rehabilitation or injury-prevention strategies that are optimized to the user’s biomechanics. In addition, IMUs are an inexpensive way to measure movement in large cohorts, facilitating large-scale multi-center clinical trials for which traditional motion capture is currently infeasible.

IMUs have been used to estimate kinematics during human movement for the past 30 years [2] and over the past decade, the biomechanics and rehabilitation communities have substantially improved the accuracy of IMU-based methods for measuring kinematics. For example, researchers have developed new sensor fusion algorithms to estimate orientations [3–6] and devised more precise sensor-to-body segment alignment methods [7, 8]. Researchers have also employed biomechanical models [9–11], and used neural networks and optimization [12–15] to estimate accurate kinematics without reliance on potentially distorted magnetometer data or precise IMU placement. Some studies have shown accuracy for lower extremity kinematics on the order of one degree root mean square (RMS) difference compared to optical motion capture. However, the overwhelming majority of studies only assess accuracy of steady-state behavior (e.g., walking or running) over short durations (on the order of one minute or less) [16], even though these conditions are not wholly representative of natural behavior.

A key challenge when estimating 3D orientation from IMUs over long durations is managing compounding drift over time. Most IMU-based algorithms to estimate joint kinematics rely on three-dimensional orientations computed through sensor fusion, a process where triaxial data from the accelerometer, gyroscope, and/or magnetometer are combined to give a more accurate measure of orientation than could be provided by any of the single data streams. Strap-down integration, or integrating gyroscope data from an IMU that is strapped to the body segment of interest (as opposed to mounted on a stabilized platform), results in random drift as numerical integration amplifies noise in the gyroscope data [17]. Accelerometer-based and magnetometer-based compensation can correct this drift using Earth’s gravitational and magnetic field vector. However, ferromagnetic disturbances distort the measurement of the earth’s magnetic field, which can lead to inaccurate orientation estimates [18]. Sensor fusion approaches have been designed to mitigate drift in specific and precise movements such as those performed by robots [6]. Validation studies applying sensor fusion methods in human movement analysis have reported average RMS differences in the range of 1.7°–8° for joint angles over short durations (on the order of one minute) [5, 19–21]. Recent work has shown that sensor fusion algorithms [22–24] can produce estimations of sensor orientations with less than 10° RMS difference over five minutes. We extend the work by estimating drift-free physiologically realistic joint angles in new experimental conditions over 10 min.

Previous studies have computed IMU-based estimates of kinematics using biomechanical models with accuracies under 5° RMS difference. These results suggest the physiological joint constraints of biomechanical models may mitigate errors due to drift. For the most part, however, these studies have used closed-source commercially available models (e.g., MVN Xsens) [25] that cost on the order of $10 k, or simple models that are developed in-house and thus are limited to users with IMU and modeling expertise [10, 26–30]. Tagliapietra et al. (2018) provide an open-source IMU-based inverse kinematics algorithm using a biomechanical model; this study reports good agreement (RMS differences less than 6 degrees) between their IMU-based estimates of kinematics and the robotic-encoder-based or optical-based kinematics, but the approach has not been tested for human movement.

Ideally, the research community would have access to an open-source platform that allows computation of kinematics from experimentally recorded IMU data using a physiologically representative musculoskeletal model that has been evaluated for use over long durations. This integrated environment would empower researchers to generate further analyses and insights (e.g. estimations of musculotendon lengths or velocities required to generate motion) that would otherwise involve invasive and complex experiments.

Our goal was thus to develop an open-source workflow for computing three-dimensional joint kinematics with IMU sensors using a human musculoskeletal model that was accurate and capable of assessing and mitigating drift. To evaluate our workflow, we compared against optical motion capture data during 10-min periods of common activities. To facilitate access of long-duration validation data to the research community, we also sought to provide an open dataset of synchronized IMU and optical motion capture data.

## Methods

### Data collection

We collected IMU and optical motion capture data for 11 subjects in a laboratory environment, which included significant amounts of electronic equipment and ferromagnetic materials. All subjects provided informed consent to a protocol approved by the Stanford University Institutional Review Board. Subjects were young (27.9 ± 6.7 years, mean ± 1 standard deviation (sd) and free of any musculoskeletal injuries or disorders; the mean body mass index of subjects was in the “normal” range (23.7 ± 2.4 kg/m^2^) and the majority were male (9/11). Subjects were outfitted with 8 IMUs (MTw Awinda, Xsens North America Inc., Culver City, USA), which were affixed to thin plexiglass plates with clusters of at least 4 retro-reflective markers, constituting a *marker plate*, and secured to the upper back (T2), lower back (L5), and the right and left thighs, shanks, and feet (Additional file 1: Figure S1). IMU signals were sampled at 100 Hz for nine subjects, and at 40 Hz for two subjects (due to a protocol inconsistency). IMU data were acquired via a graphical interface (MT Studio, Xsens North America, USA).

Optical motion capture data were collected simultaneously to enable comparison of the IMU-based estimates of joint kinematics to the current gold standard. In addition to the markers on the marker plate, markers were placed on the bony landmarks of the C7 vertebrae, sternoclavicular joints, acromion processes, anterior and posterior superior iliac spines, medial and lateral femoral epicondyles, medial and lateral malleoli, calcanei, and 5th metatarsal heads. Markers on the medial femoral epicondyles and malleoli and makers obscured by the marker plates were removed prior to walking trials. Marker trajectories were measured at 100 Hz using an eight-camera motion capture system (Motion Analysis Corporation, Santa Rosa, CA, USA). A standard video camera (30 frames/s) was used to record each trial and visually confirm events or event timings offline. The optical motion capture and IMU data were synchronized by maximizing the cross-correlation between the resulting joint kinematics.

### Experimental conditions

Experimental data were collected while each subject completed two conditions: (i) 10 min of walking and turning and (ii) 10 min of a repeated series of movements. Subjects started each condition with an initial calibration pose, standing with their arms by their sides, feet hip’s width apart, and facing forward for a period of 5 s. In the first condition, subjects were instructed to walk straight for 5 m at a self-selected pace then turn 180° using a self-selected strategy and to repeat this sequence for a continuous 10-min trial. Next, subjects took the calibration pose again, and then completed multiple cycles of lower-extremity movements for 10 min. Each cycle consisted of sitting, standing, ascending and descending three stairs, side-stepping for five meters, walking around a 12-m oval circuit, and finally running around a 12-m oval circuit. Subjects completed the cycle 6–10 times over the 10 min.

### Sensor fusion

We tested three sensor fusion algorithms: a proprietary filter (embedded on-board the Xsens IMU sensor), and two open-source complementary filters [3, 5]. The complementary filters used the raw accelerometer, gyroscope, and magnetometer signals read from the IMU sensors. We implemented the complementary filters using the developers’ open-source code [3, 5] in MATLAB R2019a (Mathworks, Inc., Natick MA, USA) with the initial orientation estimate computed from the accelerometer and magnetometer measurements when the sensors were at rest (i.e., when the subject was standing still for a few seconds). We manually tuned the filter gain (“beta parameter”) of the complementary filters [5] using data from two randomly chosen subjects (sampled at 100 Hz), and this filter gain of 0.1 was used for all subjects with data collected at 100 Hz. For the two subjects with data collected at 40 Hz, the filter gain value of 0.1 overcorrected drift, resulting in poor accuracy. Therefore, we experimented with filter gains of 0.05 and 0.025 and found the latter was optimal. To evaluate the accuracy of the sensor fusion algorithm, we compared the IMU-based orientation estimates to those computed using the motion capture markers affixed to the marker plates. The orientation difference was expressed as the angle of an axis-angle representation of the relative rotation. From this angle, we computed RMS errors. As the markers and IMUs were rigidly mounted to the marker plate, we would expect minimal errors in the optical estimates of orientation, thus we report RMS errors and refer to them as *sensor fusion errors.*

### Inverse kinematics workflow

We used OpenSim 4.2 [31, 32] (simtk.org/projects/opensim) to compute both IMU-based and optical-motion-capture-based estimates of kinematics, which we refer to as IMU-based kinematics and optical-based kinematics, respectively. We used a physiological skeletal model with 22 segments and 43 degrees-of-freedom (dofs). The model had 16 dofs in the lower body including 6 for the pelvis and 5 for each lower extremity. The hip was modeled as a ball-and-socket ﻿joint (3 dofs), the knee as a custom joint with 1 dof [33–35]. The ankle and foot in the model of Rajagopal and colleagues [36] was simplified to a single pin joint representing ankle plantarflexion-dorsiflexion, with the subtalar and mtp joints removed by welding (making them rigid). The model was scaled to match each subject’s anthropometry based on experimentally-measured markers placed on anatomical landmarks. Model scaling was only relevant for computing optical-based kinematics, as rigid body segment length does not affect IMU-based inverse kinematics. For optical-based kinematics, we used an inverse kinematics algorithm to solve for the joint angles that minimized the difference between the experimentally measured marker positions and the corresponding virtual markers on the model.

Our OpenSense toolkit in OpenSim 4.2 was used to compute IMU-based joint kinematics. The IMU orientation data resulting from a given sensor fusion algorithm were imported and associated with a rigid body (e.g., pelvis) based on a user-defined sensor mapping. To determine the orientation of the IMUs relative to the body segment on which they were placed, we used the calibration pose data. We used the optical motion capture data to pose the model (given that our main focus was assessing drift) and the IMU calibration data to compute the orientations of the IMUs relative to the posed model’s body segments as fixed rotational offsets. The modeled virtual IMU frames were then assigned these offsets relative to the underlying rigid body.

After this calibration step, we used Eq. 1 to compute the difference, expressed as the angle (${\theta }_{i}$) of the axis-angle representation between the experimentally measured IMU orientations (a rotation matrix expressed in Earth’s reference frame *N,* ${}^{N}{R}_{i}^{exp}$), and the orientations of the model’s virtual IMUs (${}^{N}{R}_{i}^{vir}$). We denote this difference as ${R}_{i}$. We used an inverse kinematics algorithm (Eq. 2) that solved for the joint angles (*q*) that minimized this weighted-squared difference (${w}_{i}{\theta }_{i}^{2}$).

1 $${\theta }_{i}={\mathrm{cos}}^{-1}\frac{\mathrm{tr} {R}_{i}-1}{2}, \text{  where  }\, {R}_{i}={({}^{N}{R}_{i}^{exp})}^{T}{}\, ^{N}{R}_{i}^{vir} \text{  and  }\, i \in \mathrm{IMUs}$$

2 $$\underset{q}{\mathrm{min}}\sum_{i \in \mathrm{IMUs}}{w}_{i}{\theta }_{i}^{2}$$

We used ${\theta }_{i}$ to quantify the RMS differences between the experimentally measured IMU orientations and the virtual IMU orientations. From here we refer to these RMS differences as *inverse kinematics orientation differences.* In the inverse kinematics algorithm, we downweighted the terms corresponding to the distal IMUs (reduced the relative weighting on the shank IMUs and the foot IMUs, i.e. *w*_*shank*_ and *w*_*fo*ot_) to minimize the influence of the IMUs that were closer to the in-ground metal force-plates (see Additional file 1: Table S1).

### Assessing IMU orientation data and joint kinematics

As noted above, the data were collected in a laboratory environment with ferromagnetic disturbances which resulted in distortions in IMU orientation estimates, especially in the heading direction. These erroneous IMU orientation estimates led to exaggerated hip adduction, hip rotation, and ankle flexion in the downstream inverse kinematics solution. To address this, we developed a pre-screening process that used the kinematic constraints of the skeletal model to identify experimental sensor orientations that were physiologically unrealistic. This pre-screening was part of the general pipeline, and we recommend applying it to achieve comparable results. The pre-screening process was based on the inverse kinematics orientation differences described above and did not assume knowledge of the true IMU orientations. The pre-screening process was: (i) if the differences exceeded a threshold of 45 degrees in the first 10 s of the trial, indicating poor tracking of the IMU orientation, then these data were excluded or (ii) if the average range of the difference over 60 ms bins (in the first 10 s of the trial) exceeded a threshold of 30 degrees, indicating unrealistic variability and therefore poor data, then these data were excluded. We share a subject information table indicating which IMUs were included in our analysis of joint kinematics (Additional file 1: Table S2). The values of 45 degrees and 30 degrees were chosen based on the data of five trials and kept constant for all 22 trials in the study. Seven of 11 subjects had at least one IMU excluded from analysis as a result of the pre-screening.

### Statistics

We compared the joint angles computed with IMU orientations to the joint angles computed from optical motion capture using root mean square (RMS) difference. We calculated a Pearson correlation between each subject and joint angle and reported the mean and standard deviation for the correlation coefficients and average change in correlation coefficients over 10 min (Table 1). Bilateral joint measures were pooled for all summary statistics. As some data were not normally distributed, as determined by a Shapiro–Wilk test, we computed the median and interquartile range of RMS difference over all subjects and all minutes for each joint angle. Outliers were defined as values 1.5 times the interquartile range below or above the 25th and 75th percentile (corresponding to the bottom or top of the box, respectively, in box plots).

**Table 1:** Correlation coefficients between IMU- and optical-based kinematics over 10 min of overground walking, averaged over all subjects

Joint angle	Overall correlation (r)	Average difference in correlation (r) between 1st and 10th minute
Pelvic tilt	0.60 (0.30)	0.1 (0.1)
Pelvic list	0.65 (0.23)	0.002 (0.1)
Hip flexion	0.84 (0.29)	− 0.002 (0.01)
Hip adduction	0.60 (0.27)	− 0.002 (0.06)
Hip rotation	0.71 (0.27)	− 0.03 (0.04)
Knee flexion	0.87 (0.32)	− 0.003 (0.04)
Ankle plantarflexion	0.70 (0.10)	− 0.1 (0.1)

Mean (standard deviation)

We quantified drift for each subject and joint angle using a linear regression on each subject’s individual per-minute RMS differences for each joint angle over the 10 min. For each joint angle, we averaged the slopes of the linear fits across subjects. We report the range of slopes to represent drift as an average change in RMS difference per minute. To examine changes over time in RMS differences for different sensor fusion algorithms, we subtracted a subject’s joint angle RMS difference at the end of the first minute from the RMS difference at the end of the 10th minute. We completed all statistical analyses in MATLAB R2019a.

## Results

**Fig. 1:**
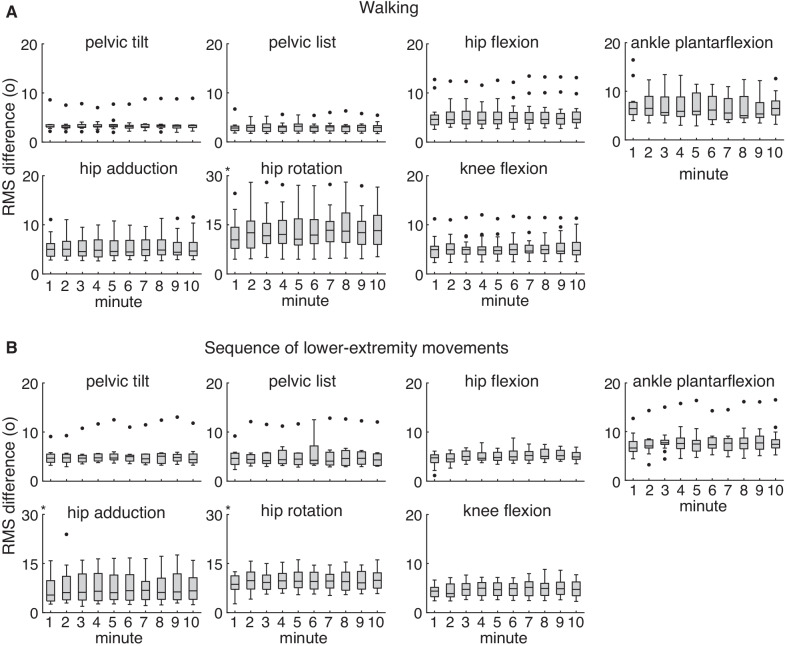
Root mean square (RMS) differences for IMU-based lower extremity joint kinematics over 10 min. Our open-source workflow produced IMU-based kinematics comparable to optical-based kinematics during **A** a 10-min period of overground walking and **B** a 10-min sequence of common lower-extremity movements. Median RMS differences between IMU and optical-based kinematics were 3–6° for all joint angles except hip rotation (12°) over all subjects and all minutes. Flat trends across median per-minute RMS differences highlight minimal drift over 10 min. Box plot height is equal to interquartile range with outliers (black dots) defined as values exceeding 1.5 times the interquartile range. The asterisk denotes a different y-axis range. Results shown used the complementary filter [5]

Median RMS differences between IMU and optical-based kinematics were 3–6° over all subjects and all minutes (Fig. 1) for all joint angles except hip rotation (12°); these values are within the reported variability and uncertainty of optical motion capture [37]. We saw a similar range of RMS differences between IMU and optical-based kinematics for the 10-min sequence of lower-extremity movements. Results for the two open-source complementary filters were similar, so we focus on the results produced with the open-source algorithm from Madgwick et al. [5] and refer to it as the “complementary filter”. Minute-by-minute RMS differences for the complementary filter from Mahony et al. [3] can be found in Additional file 1: Figure S2 and highlight that these trends for RMS differences over time were also largely flat.

Lower extremity joint kinematics showed minimal drift. Median RMS differences were largely unchanged over 10 min for all joint angles during both conditions (Fig. 1). Each individual subject’s per-minute RMS differences showed minimal change over 10 min for all joint angles (Additional file 1: Figure S3), and the average slopes of the linear fits to these data were near zero (− 0.14–0.17°/min), indicating minimal drift. Though the linear fits were not strong (R^2^ = − 0.03–0.4), this was likely due to the nearly horizontal trajectories of the subjects' RMS differences, at which point R^2^ approaches a negative value (i.e., the chosen model fits worse than a horizontal line).

**Fig. 2:**
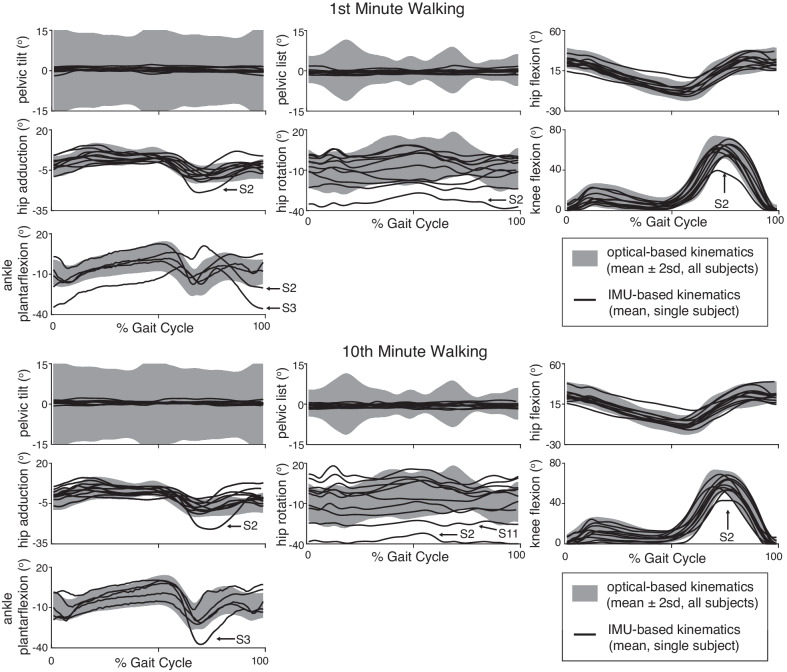
IMU-based lower extremity joint kinematics in the 1st minute (top) and 10th minute (bottom). Individual subjects’ IMU-based kinematics for the right side of the body during the 1st and 10th minute of overground walking (N = 10 subjects, one subject, S1, lacked any periods of straight-walking and was omitted from this plot). Mean ± two standard deviations (sd) for optical-based kinematics is shown as a grey shaded band and individual subject means for IMU-based kinematics are shown as black lines

Individual subjects’ mean IMU-based joint angles over the gait cycle showed minimal difference (within two standard deviations) from optical-based joint angles between the 1st and 10th minute of overground walking (Fig. 2). IMU-based hip rotation showed the least agreement with optical-based kinematics. A few subjects (Subjects 2,3,11) had IMU-based kinematics outside two standard deviations of optical-based kinematics (Fig. 2). The residual plots between the IMU-based and optical-based kinematics for all subjects are included in Additional file 1: Figure S4.

We found moderate to strong correlations between the IMU-based kinematics and the optical-based kinematics as indicated by high correlation coefficients over the 10-min period of overground walking, ranging from r = 0.60–0.87 (Table 1). The average difference in correlation coefficient between the first and 10th minute was also near zero (− 0.1–0.1), indicating little change or drift over the 10 min. The results were similar for the sequence of lower-extremity movements (Additional file 1: Table S3) and also whether we used the complementary filter or the proprietary filter from Xsens (Additional file 1: Figure S5).

Joint angles computed using the complementary filter and the proprietary filter from Xsens showed similar changes in median RMS difference over 10 min, with less than 2 degrees versus less than 4 degrees, respectively, during walking (Additional file 1: Figure S5). The proprietary filter, however, had more and larger outliers than the complimentary filter. For example, almost 50 degrees change in RMS difference in knee flexion was recorded for one subject (Additional file 1: Figure S5), highlighting that when the proprietary filter starts to drift, errors can accumulate quickly and substantially. Similar results were achieved for the sequence of lower-extremity movements (see minute-by-minute RMS differences in Additional file 1: Figure S6).

**Fig. 3:**
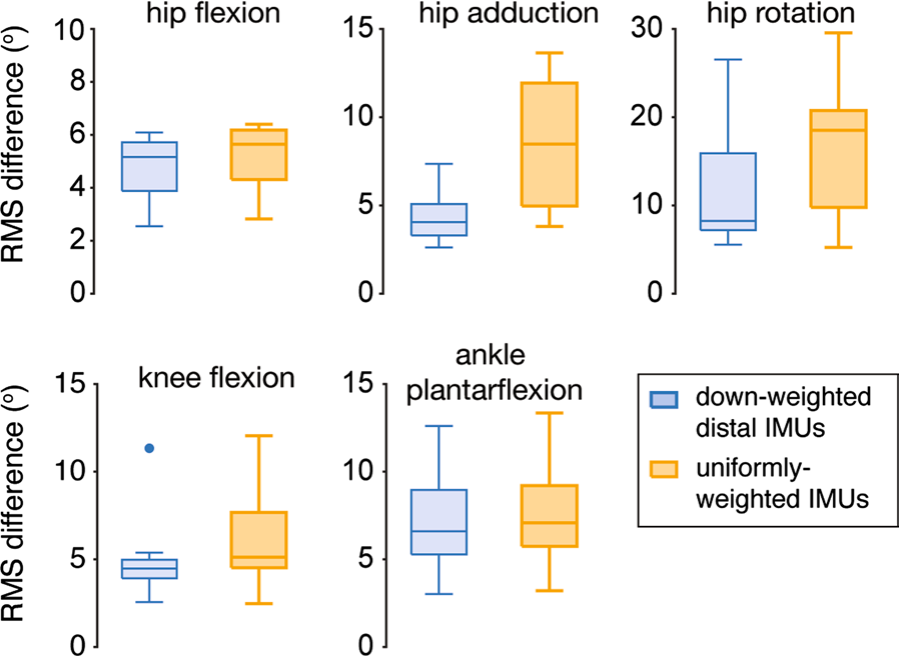
Effect of downweighting distal IMU sensors when solving inverse kinematics. Reducing the relative weighting on the shank orientations and the feet orientations when solving inverse kinematics helped reduce mean joint angle root mean square (RMS) difference in the 10th minute. To highlight how this downweighting influenced all joint kinematics, this analysis included mean joint angle RMS differences for the four subjects who did not have IMUs excluded and results computed from the complementary filter

We found that downweighting the distal sensor orientations (reducing the relative weighting on the shank and feet sensor orientations) when solving inverse kinematics improved the accuracy of the kinematic estimates and reduced drift. The inverse kinematics computed with downweighted distal sensors’ orientations showed less RMS difference (up to 28% less) than inverse kinematics computed with uniformly weighted orientations in the 10th minute of overground walking (Fig. 3). Note that all other figures with IMU-based kinematics show downweighted results.

**Fig. 4:**
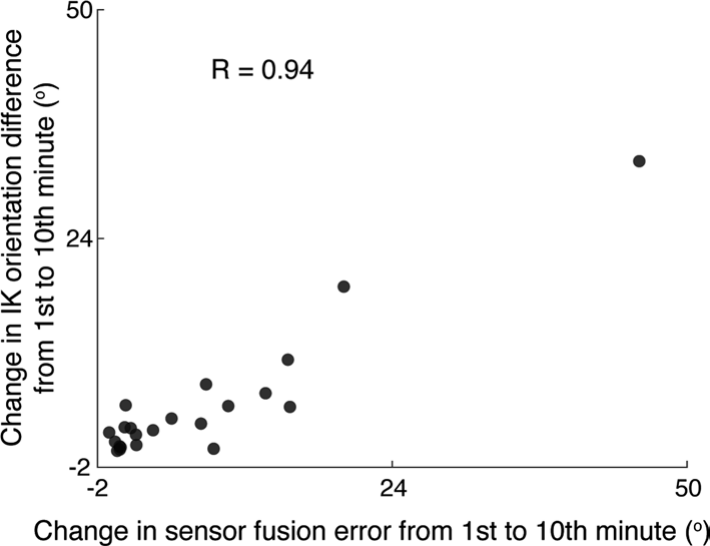
Changes in inverse kinematics (IK) orientation differences relate to changes in sensor fusion errors. Changes in IK orientation differences (mean over all joint angles per subject) from the 1st to 10th minute were strongly correlated with changes in sensor fusion error, indicating that IK orientation differences are a helpful tool for tracking error in the sensor fusion orientation when present. Individual subjects’ data are represented by black circles, and kinematics computed with both the complementary and the proprietary filter were used

Changes in inverse kinematics orientation differences (the angle difference between the experimental IMU orientation and the virtual IMU orientation) from the 1st to 10th minute were strongly correlated with changes in sensor fusion error (Fig. 4), indicating that inverse kinematics orientation differences are a helpful tool for tracking errors in the orientations from sensor fusion when present.

## Discussion

Our open-source workflow for computing three-dimensional lower extremity joint kinematics with IMUs produced joint angles that were consistent with optical motion capture (3°–6° RMS differences for all joint angles except hip rotation) and showed minimal drift during a 10-min period of common lower-extremity movements (including walking). The differences between IMU-based kinematics and optical-based kinematics are similar to previous studies [16], despite our experiments being an order of magnitude longer in duration. We also found that using sensor fusion approaches, as well as downweighting distal IMUs during inverse kinematics, mitigates drift during these common lower-extremity movements. Our open-source workflow, documentation, data, and models are shared at https://simtk.org/projects/opensim and https://simtk.org/projects/opensense_val so that others can reproduce and extend our work.

Our results suggest that explicit sensor recalibration (e.g., sitting or standing still for a few seconds or zero velocity updates) may not always be necessary to mitigate drift when monitoring human movement with IMUs. Further study is needed to determine whether this also occurs under more general experimental conditions and outside of the laboratory. Our workflow leveraged complementary filters [3, 5] that incorporate magnetometer data. Our study indicates that natural human movement—even continuous walking without explicit periods of rest—includes phases where each individual body segment has a low angular velocity. During these phases, the sensor fusion algorithms were able to reject drift. These implicit corrections by the sensor fusion algorithm occurred at different times depending on the activity, but for each of the movement trials (continuous walking and a sequence of movements), the frequency and the duration of periods of low angular velocity were sufficient to mitigate drift. The approach further has the benefit of being activity-agnostic, compared to some previous approaches that were tailored to specific activities (e.g., [38] for running; [39] for specific phases during walking) or reliant on achieving relatively high joint center accelerations [40, 41]. In our computational experiments, the RMS differences during the first 15 s of walking were similar between our sensor fusion estimate and an estimate obtained by solely integrating the angular velocities over time (Additional file 1: Table S4), indicating that the drift correction in the sensor fusion did not significantly degrade accuracy over short durations. In this work, we used a linear regression to show that drift did not increase over our 10-min trial. An analysis of how errors accumulate over short periods of time (i.e., where the linear analysis might be inadequate) would be a valuable topic of future study.

The proprietary filter included with the sensors resulted in more drift for all subjects (Additional file 1: Figure S5), likely because the filter rejected the distorted magnetometer signal over too long a duration. Recent work has shown that Kalman filters can be formulated to achieve good accuracy over long durations [24]. Some prior work has shown complementary filters drift with measurement duration [23, 42]. We suspect that these studies have used different sensor fusion parameters than those reported in our study and these parameters were critical to achieve the drift-free orientation estimation. Future work should explore a variety of sensor fusion algorithms and whether they are sufficiently robust for periods of continuous running, sprinting, or other highly dynamic activities, as well as activities outside of the laboratory.

The biomechanical model and inverse kinematics algorithm used in our workflow (OpenSense, OpenSim), provided features that helped us to monitor errors when they did occur. For example, we saw that a change in inverse kinematics orientation differences from the first to the 10th minute was strongly correlated with sensor fusion error (Fig. 4) demonstrating utility for monitoring joint angle accuracies. We also used the inverse kinematics orientation differences to screen for IMUs that presented large differences early in the experiment (within the first 10 s). Monitoring inverse kinematics orientation differences could alert users to the presence of error, as knowledge of the true IMU orientations is not required. This is a salient feature because there is currently no standard method to monitor error over time for IMU-based kinematics. As the reliance on IMUs for quantifying human movement experiments continues to increase, users will benefit from having error-monitoring features integrated with user-friendly software (OpenSim) that possesses a significant community of 25,000 + users/year worldwide [31]. Users can extend these tools to meet their research needs by combining IMU-based estimates of motion with a range of open-source models or explore underlying quantities like muscle dynamics.

Our study also provides insights about how different joints are affected by magnetic disturbances and drift. For example, while magnetic disturbances can cause large errors in heading angles, our results suggest that these errors do not significantly impact the accuracy of the knee flexion angle. This is an important observation as a major concern in the adoption of IMUs in biomechanics and physical rehabilitation is the presence of magnetic disturbances in the patient’s home. We suspect that knee flexion accuracy is not severely impacted by magnetic disturbances because these disturbances will likely impact the heading of both the shank and the thigh similarly, and possibly because of the kinematic constraint on the knee (i.e., one dof in the sagittal plane). We observed that the IMU orientations computed from sensors on the feet were most affected by magnetic disturbances. In laboratories similar to ours with in-ground force plates, researchers have reported significant magnetic disturbances to the point where they recommended that measurements be performed at least 40 cm off the ground [18]. We were able to increase the agreement between IMU- and optical-based kinematics by reducing the frequency at which the complementary filter incorporated magnetometer data for the foot IMU (Additional file 1: Figure S7). This balanced the positive influence from some magnetometer information and distortion from the force plates. As this may be overfitting to our data, we did not use this approach when reporting our final results, but offer it as an approach to explore further when computing IMU-based kinematics in disturbed magnetic environments.

Similar to past studies, we saw the largest joint angle RMS differences in hip rotation (12.7 degrees median RMS difference). This could be partially due to the fact that the foot IMUs were most affected by ferromagnetic disturbances, and their distorted heading estimates could have introduced error into the hip rotation angle, which was the dof that relied most on this heading information. RMS errors on the order of 10 degrees are also observed for optical motion capture, which may also have contributed to the RMS differences we observed [37]. We also qualitatively observed large hip adduction errors due to magnetic disturbances while the subjects were sitting in the sequence of lower-extremity movements. Future work is needed to assess joint-specific impacts of magnetic disturbances.

It is important to consider the limitations of our work. We used the optical motion capture data to pose the model for IMU registration, which is an unrealistic approach for natural environments. We instructed subjects to take a “neutral” pose, with pelvic tilt, pelvic list, hip flexion, hip adduction, hip rotation, knee flexion, and ankle flexion at 0 degrees. The mean difference between the subject’s chosen pose (measured with motion capture) and the instructed pose was relatively small (3.8°), and the difference range was 0–20.1 degrees (maximum was hip rotation) over all joint angles, and all subjects. A range of calibration approaches including manual, static, functional, and anatomical methods have been described and assessed [7]. While using an IMU-based calibration might have altered the RMS differences, we expect our conclusions showing minimal drift would be unchanged.

Despite being one of the longer validation studies for IMU-based kinematics, our 10-min experiments may not be sufficient to understand the accuracy over multiple hours or days. However, we have three cases where the IMUs were calibrated between seven and twelve minutes before the start of the 10-min experiment and we again observed minimal drift (Additional file 1: Table S5). This suggests that our results might translate to longer durations, but future experiments should assess this. In this study, we focused on assessing the accuracy over an aggregate of activities. Future work should validate the approach for the individual activities in the sequence of lower-extremity movements, along with other activities of daily living, including upper body kinematics, and more natural sequences of these activities. Since our sample size was small and our subject demographics were homogenous, it is uncertain how our results may translate to other populations. We hope that future studies will be conducted with larger, more diverse populations and will build upon the data repository we have provided.

## Conclusions

Our open-source workflow (OpenSense, OpenSim) provides accurate estimates of human joint kinematics with wearable technology by leveraging the advantages of inertial sensors, sensor fusion algorithms, and model-driven simulation. The validation over 10-min durations during common human movements gives confidence to users in being able to monitor and compute kinematics with minimal drift using IMUs. Though all of our kinematics were calculated offline, a recent study has shown promising accuracy over short durations with a low-cost and portable system utilizing the same open-source tools to compute inverse kinematics described here (42). Integration with the OpenSim musculoskeletal simulation environment opens the gateway to investigate other quantities, like muscle mechanics and inverse dynamics with IMUs. Future work will focus on developing algorithms to estimate kinetics from IMU data and streamlining real-time systems to enable biomechanical monitoring, feedback, and interventions outside of the lab. This suite of open-source tools brings the field closer to conducting human movement studies in natural environments.

## Supplementary Information

**Additional file 1:** Supplementary Information, Figures S1–S7 and Tables S1–S5.

## Data Availability

The dataset supporting the conclusions of this article is available in the SimTK repository. We have deposited our data at https://simtk.org/projects/opensense_val (https://doi.org/10.18735/2f9k-ca43). The software is available at: https://simtk.org/projects/opensim Project name: OpenSense, OpenSim. Project home page: https://simtk.org/projects/opensense Archived version: https://simtk.org/frs/?group_id=1715 Operation system(s): MacOS, Windows. Programming language: C +  + Other requirements: n/a. License: Apache License, Version 2.0 (the "License").

## Abbreviations

IMU: Inertial measurement unit
IK: Inverse kinematics
RMS: Root mean square
